# The Neutrophil's Eye-View: Inference and Visualisation of the Chemoattractant Field Driving Cell Chemotaxis *In Vivo*

**DOI:** 10.1371/journal.pone.0035182

**Published:** 2012-04-26

**Authors:** Visakan Kadirkamanathan, Sean R. Anderson, Stephen A. Billings, Xiliang Zhang, Geoffrey R. Holmes, Constantino C. Reyes-Aldasoro, Philip M. Elks, Stephen A. Renshaw

**Affiliations:** 1 Complex Systems and Signal Processing Group, Department of Automatic Control and Systems Engineering, University of Sheffield, Sheffield, United Kingdom; 2 School of Engineering and Design, University of Sussex, Brighton, United Kingdom; 3 MRC Centre for Developmental and Biomedical Genetics, University of Sheffield, Sheffield, United Kingdom; 4 Academic Unit of Respiratory Medicine, Department of Infection and Immunity, University of Sheffield, Sheffield, United Kingdom; University of São Paulo, Brazil

## Abstract

As we begin to understand the signals that drive chemotaxis *in vivo*, it is becoming clear that there is a complex interplay of chemotactic factors, which changes over time as the inflammatory response evolves. New animal models such as transgenic lines of zebrafish, which are near transparent and where the neutrophils express a green fluorescent protein, have the potential to greatly increase our understanding of the chemotactic process under conditions of wounding and infection from video microscopy data. Measurement of the chemoattractants over space (and their evolution over time) is a key objective for understanding the signals driving neutrophil chemotaxis. However, it is not possible to measure and visualise the most important contributors to *in vivo* chemotaxis, and in fact the understanding of the main contributors at any particular time is incomplete. The key insight that we make in this investigation is that the neutrophils themselves are sensing the underlying field that is driving their action and we can use the observations of neutrophil movement to infer the hidden net chemoattractant field by use of a novel computational framework. We apply the methodology to multiple *in vivo* neutrophil recruitment data sets to demonstrate this new technique and find that the method provides consistent estimates of the chemoattractant field across the majority of experiments. The framework that we derive represents an important new methodology for cell biologists investigating the signalling processes driving cell chemotaxis, which we label the neutrophils eye-view of the chemoattractant field.

## Introduction

There are many cell-types whose movements are driven by sensing external chemical gradients in the process known as chemotaxis [1], [2]. For instance, in response to tissue damage and infection resulting from wounding, neutrophils are recruited to the site of injury guided by chemoattractants [3], [4]. Neutrophils are a key component of the body's immune system, responding rapidly to bacterial incursions, sterilising microbial pathogens and working cooperatively with other cells of the immune system (e.g. macrophages) to resolve infections and then switch from a pro- to an anti-inflammatory state [5], [6]. There has been recent progress on representing our knowledge of chemotaxis in neutrophils and eukaryotic cells in mathematical models, for instance in gradient sensing [7], pseudopod formation [8], [9] and cell polarization [10]. However, there are still many open questions regarding the complex signalling processes that drive neutrophil migratory responses [11], which are now being increasingly studied *in vivo* [12]–[14]. Since targeting chemotaxis is a potential way to reduce the neutrophil burden in inflammatory disease, visualising the process *in vivo* and using mathematical modeling approaches on the data obtained should provide new insights, with the ultimate goal of developing new therapeutic approaches for treating unwanted inflammation.

In the past few years, powerful techniques based on transgenic animal models have emerged that allow us to view neutrophil migration to a wound *in vivo*, such as the zebrafish model (*Danio rerio*), where neutrophils are labelled with a green fluorescent protein (GFP) [15], [16]. The near transparency of zebrafish larvae, in conjunction with GFP-labelling of the neutrophils, facilitates observation and recording of neutrophil movement by videomicroscopy. The use of this technique gives us new opportunities to study neutrophil recruitment and inflammation resolution as caused by natural processes of chemical signalling, which may provide important insight into, for instance, the role of neutrophils in respiratory disease [17], [18].

One challenge that *in vivo* experiments present, in comparison to *in vitro* studies of neutrophil responses to a highly regulated chemical gradient [19]–[21], is the identification of the underlying chemoattractant field, which is unknown and not controlled (by the investigator). Whilst it is possible to image specific chemicals that might be acting as signalling agents [22], the direct observation of the net field (or simultaneous observation of all signalling agents) driving neutrophil movement is likely to be always beyond reach. This problem motivates the development of methods for chemoattractant field identification, not from direct measurement, but from functionally related variables such as neutrophil movement.

From video recordings of neutrophil action, their response to the surrounding chemoattractant field driving their movements can be observed, although that field itself remains hidden from view. The question therefore arises - can we infer the underlying chemoattractant field from observations of the cell movement? If this were possible, we could then see the chemoattractant landscape from the perspective of the neutrophil itself - a neutrophil's *eye view* of the chemoattractant field, providing insight into the guidance cues directing their movement.

This type of problem is one typically encountered in signal processing, where a hidden variable of interest must be inferred from functionally related observations [23], [24]. Here, we pose the question: what is the function that maps from the observed signal to the hidden variable - from the cell movement to the chemoattractant field? In this study, we create a novel framework for estimating and visualising the chemoattractant field based on a simple assumed relationship between cell movement and field. Motivated by the Keller-Segel model of chemotaxis [25], [26] we assume that cell velocity is proportional to the chemoattractant gradient. From this assumption we derive an identification scheme using a multiscale basis function decomposition [27], [28] of the chemoattractant field combined with a Bayesian approach to parameter estimation [29]. This data-driven inference framework is contingent on the availability of cell velocity estimates over space, and therefore requires an informative set of cell tracks. Hence, the quality of the derived model is directly linked to the information contained in the observations of cell movement.

**Figure 1:**
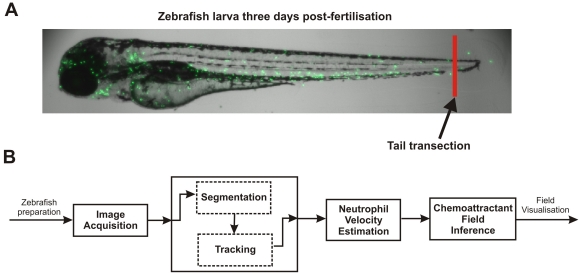
Zebrafish experimental setup and neutrophil analysis procedure. A: Zebrafish larva from the transgenic line, Tg(mpx:GFP)i114. Neutrophils are visualised by excitation of green fluorescent protein, as previously described (Renshaw et al., 2006). The zebrafish were prepared by transection of the tailfin at the site indicated to elicit an inflammatory response, which caused recruitment of the neutrophils to the site of injury. B: The chemoattractant field inference framework. Firstly, images of neutrophil recruitment to the zebrafish wound site were acquired by video microscopy. The neutrophil centroid positions were then obtained from a segmentation and tracking algorithm. Velocities of the neutrophils were estimated from the neutrophil centroid tracks using a Kalman smoother and lastly, the velocity estimates were used in the inference of the chemoattractant field.

In order to investigate the chemoattractant field inference framework we applied the technique to (i) an *in vitro* dataset of human neutrophils responding to interleukin-8 [30] and (ii) to a number of datasets (n = 15) of neutrophil recruitment *in vivo* in the zebrafish. The *in vivo* observations of cell movement were obtained using confocal video microscopy from a transgenic line of zebrafish [15]. GFP-labelling of cells facilitated the process of segmentation and tracking: we used a specially designed neutrophil tracker to obtain cell tracks in terms of centroid positions [31]. Position tracks were then used to derive velocity estimates of the cells by a signal derivative estimation algorithm [32], which made use of the Kalman smoother state estimator [33]. Neutrophil velocity estimates were used to drive the field inference algorithm (the full procedure is summarised in Figure 1). The resulting data provide novel insights into the *in vivo* characteristics of the field driving neutrophil movements, and demonstrate a powerful new technique for estimating and visualising the chemoattractant landscape from the perspective of the cell.

## Results and Discussion

### Velocity of neutrophils in the cell recruitment phase

**Figure 2:**
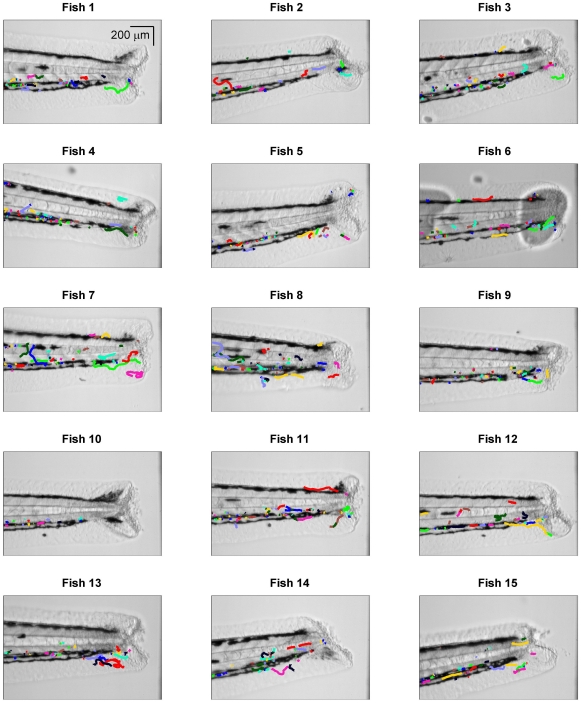
Neutrophil centroid position tracks. The neutrophil tracks (colour lines) were obtained from a segmentation and tracking algorithm and are shown here in relation to the zebrafish image (greyscale), where the zebrafish image of dimension 1000
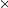
1000 pixels has been zoomed on the vertical axis to the 100–900 pixel range.

Zebrafish were prepared and imaged from 30–60 mins after injury as described in Methods. The tracking algorithm (Methods) was used to segment and link cell positions across video frames, and from the cell positions the centroids were extracted to form tracks. The cell tracks were typically spatially distributed either side of the notochord, around vascular structures, presumably due to the physical characteristics of the local environment (Figure 2). The tracks tended to cluster either above or below the notochord and to lie in a relatively narrow space along the dorso-ventral axis (e.g. Fish 1 in Figure 2). This might relate either to physical factors of the anatomical location or to the action of early recruited cells passing through the tissue easing the passage of subsequent cells, creating a preferred pathway for movement to the wound.

**Figure 3:**
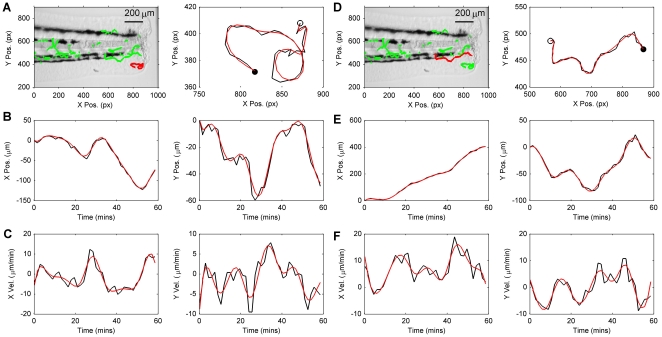
Typical examples of neutrophil tracks and neutrophil velocity estimates. A and D: The image on the left shows a highlighted red track that is zoomed in the plot on the right, in which the centroid positions extracted from the tracking algorithm (black) and smoothed track estimate (red) are compared (the open circle indicates the track start point and the filled circle indicates the track end point). B and E: X-Y cell centroid position estimates corresponding to tracks highlighted in A and B are shown as signals with respect to time produced by the tracking algorithm (black) and estimates from the smoothing algorithm (red). C and F: X-Y velocity estimates (raw estimates in black and smoothed estimates in red), corresponding to position signals in B and E. Raw estimates of velocity were obtained by numerical differencing (central difference method) applied to the tracker position estimates.

**Figure 4:**
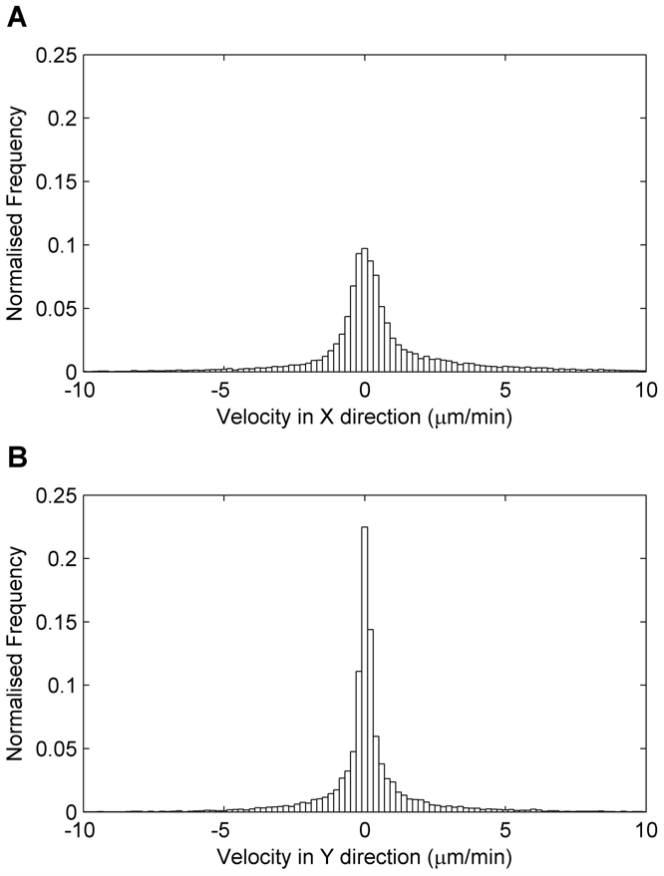
Neutrophil velocities. A: Histogram of neutrophil velocities in the X-direction at each sample time (histograms are zoomed to the −10 to 10 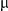
m/min range for an effective visualisation and data are aggregated over all fish). B: Histogram of neutrophil velocities in the Y-direction.

From observations of cell centroid position we estimated cell velocity by use of a Kalman smoothing algorithm (see Methods). In order to validate the velocity estimation algorithm, smoothed position and velocity estimates were compared to their raw signal counterparts. Raw signals in this case correspond to the output of the tracking algorithm for position, and central differencing applied to the raw position estimate for velocity (Figure 3). It is evident from inspection of the example tracks shown in Figure 3 that the smoothing algorithm does not distort the underlying track but rather smooths high frequencies from the position and velocity signals, which are likely due to noise in the case of position, and high frequency noise amplification due to differencing in the case of velocity. The velocities of cells were typically in the range −10 to 10 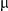
m/min and the distribution of velocities were peaked around 0 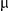
m/min (Figure 4A–B). The higher peak around 0 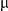
m/min in the Y-direction velocity histogram compared to the X-direction was probably due to the more active movement of neutrophils in the X-direction, corresponding to neutrophils travelling towards the wound from the anterior end.

The primary result of using the smoothing algorithm for velocity estimation was a set of velocity signals pertaining to each fish, suitable for use in the chemoattractant field inference framework.

### Inference of the chemoattractant field from observations of cell movement

**Figure 5:**
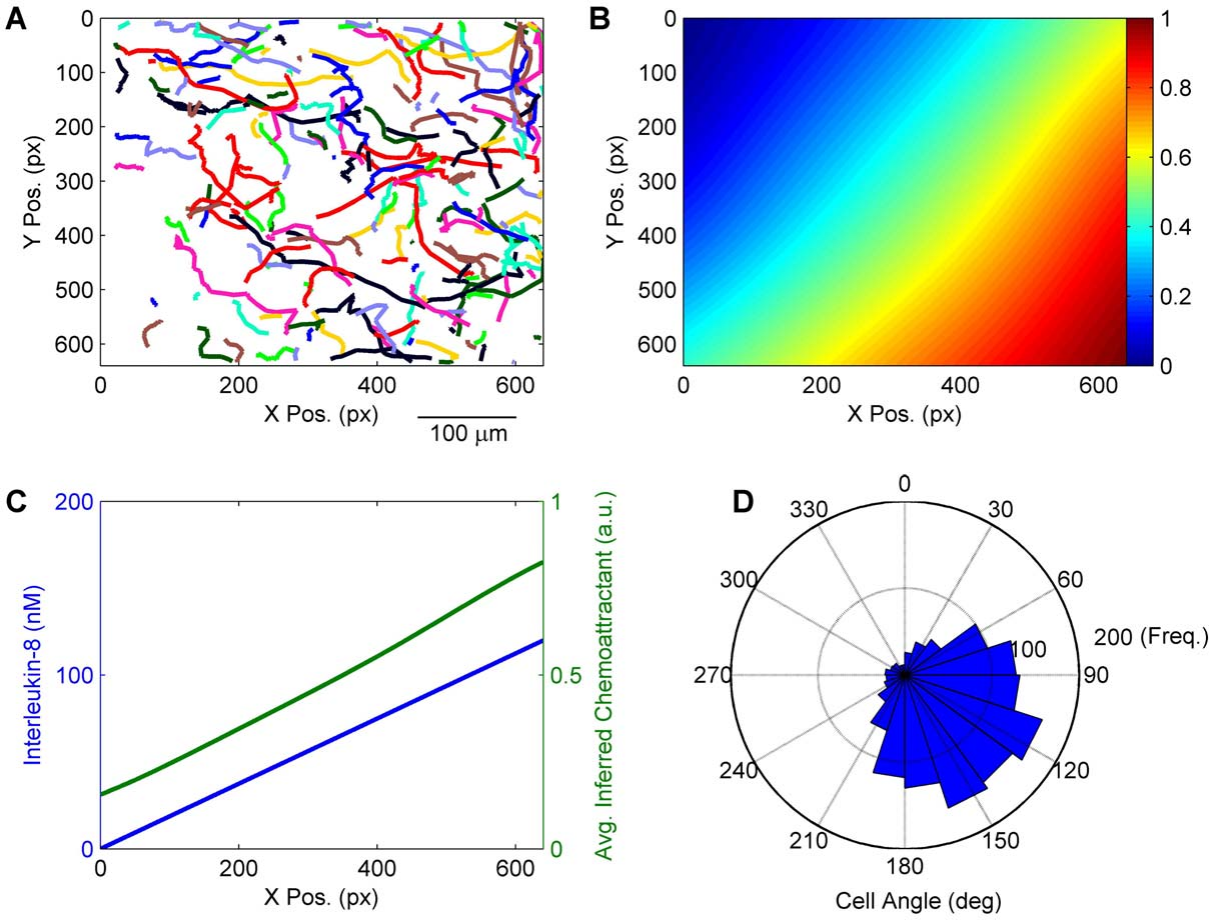
Chemoattractant field inference *in vitro*. A: Cell tracks of human neutrophils *in vitro* chemotaxing due to presence of the chemokine interleukin-8, which increases in concentration from left to right. B: Inferred chemoattractant field, normalised to the range (0,1). The chemoattractant field estimate is dimensionless hence the scale of the colormap is in arbitrary units (a.u.). C: Comparison of inferred chemoattractant field averaged over the Y-direction, to the level of chemokine interleukin-8 reported in [30]. D: Circular histogram of neutrophil angles, demonstrating a directional bias of the tracks shown in panel A towards the lower right corner.

The chemoattractant field inference framework (see Methods) was used to estimate the underlying chemoattractant field driving neutrophil movement. In order to provide a validation that the modelling framework was able to accurately infer the chemoattractant field, we applied the framework to an *in vitro* data set of human neutrophil chemotaxis [30]. In that study, a linear gradient of the chemokine interleukin-8 was set-up using a microfluidic generator. Neutrophil tracks from one assay are shown in Figure 5A (from video 2 of the supplementary material of [30]). The inferred chemoattractant field increased from left to right, corresponding to the reported level of interleukin-8 (Figure 5B and C). In addition, the inferred field was skewed towards the lower right corner. This can also be seen in the directional bias of the neutrophil movements (Figure 5D). We cannot comment on whether this bias in cell movement and field inference was a chance occurrence or the result of some non-linearity within the gradient. However, the evidence from the movement data demonstrates a skew, which the data-driven inference framework must reflect. Hence, the field inference is providing a view of the chemoattractant landscape as sensed and acted on by the neutrophils themselves.

**Figure 6:**
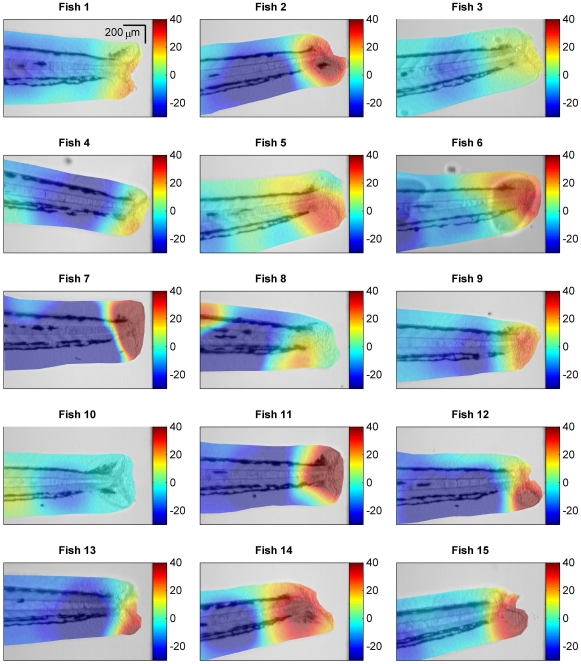
Chemoattractant field inference in the zebrafish. For each zebrafish, 1–15, the estimate of the chemoattractant field (colour) is overlayed with transparency on the fish image (grayscale). Each colormap is scaled to the range −20 to 40 to provide an effective visual comparison over all fish. The chemoattractant field estimate is dimensionless hence the scale of the colormap is in arbitrary units.

For the case of *in vivo* data the net chemoattractant landscape driving neutrophil action was not directly measurable, but was testable against the independent assumption that the field would be of higher magnitude close to the wound and weaker in regions distant from the wound. We observe from 13 of the 15 fish (Fish 1–7, 9, 11–15) that the estimated field conforms to this assumption - that the field was of higher magnitude close to the wound and decayed away from the wound (Figure 6). In the case of Fish 8 the field did exhibit a peak as expected near the wound along with a high peak towards the anterior end that can be explained by the movement of two neutrophils at the anterior end moving away from the wound with relatively high velocity. For Fish 10 we note that there was an unusually low number of cell tracks (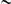
20 tracks), which appeared to be insufficient for driving the estimation framework (see Figure 2, Fish 10). These data were included in the analysis for completeness, to avoid introducing unintentional bias into the data analysis. Taken as an ensemble, the results provide consistent support for the field inference framework and the assumptions upon which it was constructed.

The chemoattractant field inference framework was derived from the assumption that cell velocity was proportional to the gradient of the field, which is a relationship described in the Keller-Segel model of chemotaxis [25], [26]. The proportionality model used here may be a simplification of the true complexity of the neutrophil movement-chemoattractant gradient relationship, however, this framework could be extended and modified in the future under modified assumptions, whilst retaining the fundamental approach. For instance, the assumption of a linear relationship between chemottractant gradient and velocity might benefit from refining at the upper extremes of the gradient range, where we might expect a nonlinear relationship, such as a saturation in velocity, to more accurately reflect neutrophil action. A key aspect of the work presented here is the initial development of a data-driven inference framework, which builds on relationships expressed through existing biological models, and demonstrates how observations of cell movement can be used to estimate the hidden field driving those cell movements.

The near transparency of the zebrafish larva, along with the ability to use genetic reporters of cell type and function, has led to the discovery of Hydrogen Peroxide gradients during wound healing [22]. These gradients are important in recruiting the first wave of neutrophils, but rapidly decline. It is striking how similar those gradients are qualitatively to those inferred here. As technology advances, it will become increasingly important to know to what degree the observed gradients match the gradient to which the neutrophils are responding, which we suggest might be achieved by comparing observations of signalling agents to the chemoattractant field inferred using the framework proposed here. In this investigation we have demonstrated that the modelling framework reflects neutrophil action *in vitro*. In future experiments, we hope to test the applicability of these approaches for known gradients *in vivo*, which more accurately reflects the complex environments neutrophils encounter in human disease settings.

We have presented the first step in visualising a static chemotactic gradient *in vivo*, and future advances will seek to address the relative importance of different chemotactic gradients as they evolve over time. Niethammer et al. [22] also show the evolution of the hydrogen peroxide gradient over time, and a key area for extending our work will be timelapse experiments that will provide analogous insight into the dynamic behaviour of the inferred chemoattractant field. This will require a description of the evolution of the spatial field over time using data-driven spatiotemporal identification techniques that are suitable for application to linear [34], [35] and possibly nonlinear [36]–[38] dynamic systems.

Furthermore, our analysis has begun as a two dimensional system, aided by the properties of the zebrafish fin, but future work in this system will allow analysis to be extended to three dimensions. This will be a particularly important advance if this is to be extended to the emerging field of *in vivo* inflammation imaging in mouse [39].

In summary, the results presented here demonstrate the effectiveness of a novel and simple-to-implement chemoattractant field inference framework, which enables visualisation of the inferred field driving neutrophil movements: a quantity that is not directly measurable.

## Methods

### Ethics Statement

All animal work was performed according to guidelines and legislation set out in UK law in the Animals (Scientific Procedures) Act 1986. Ethical approval was given by the University of Sheffield Local Ethical Review Panel.

### Image acquisition

The neutrophil specific fluorescent zebrafish line *Tg(mpx:GFP)i114* [15] was used for tracking experiments. Zebrafish strains were maintained according to standard protocols (Nusslein-Volhard, 2002). Adult fish were maintained on a 14/10 hour light/dark cycle at 28

C in UK Home Office approved facilities in the MRC CDBG aquaria at the University of Sheffield. Inflammatory responses were elicited in three days post-fertilization (dpf) zebrafish embryos by tail transection as previously described (Renshaw et al., 2006: Elks et al., 2011). Injured embryos were mounted in 1% low-melting point agarose (Sigma-Aldrich, St. Louis, MO) with 0.168 mg/ml Tricaine (Sigma-Aldrich) as an anaesthetic. Mounted embryos were imaged at one hour post-injury (hpi) on an UltraVIEWVoX spinning disk confocal microscope (Perkin Elmer Inc., Waltham, MA) using brightfield and laser excitation at 488 nm for GFP. Eight z-stacks through the whole tail thickness were acquired every 90 seconds over the timelapse period of one hour. A motorized stage on the spinning disk allowed multiple embryos to be imaged in one timelapse session. Acquisition of the timelapse images was performed in Volocity 5 (Improvision, Perkin Elmer Inc.), before exporting the data as multiple TIFF files for the analysis described below.

### Neutrophil tracking

In order to obtain tracks of neutrophil centroid positions we used a modification of a ‘keyhole’ tracking algorithm previously applied to red blood cells [31].

#### Segmentation

In the segmentation step, the raw video frames were transformed to a sequence of binary images containing segmented foreground objects (the neutrophils). Due to the large frame size of the zebrafish images (1000
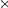
1000 pixels) a pyramid level method was used to reduce computational complexity and associated processing time [40]: at each level a group of four contiguous voxels were averaged to produce a new pixel, thereby halving the number of rows and columns of the image. In this case we used one level only, to reduce the image from 1000×1000 to 500×500 pixels. The level processing method had the added benefit of reducing noise by smoothing the raw image.

The intensity of each video frame was thresholded using a hysteresis method, where voxels below a lower threshold were classified as background and those above a higher threshold were classified as neutrophils. The remaining voxels, between these two levels, were then classified as neutrophils if they were in contact with voxels above the high threshold or background otherwise. Both thresholds were automatically determined using Otsu's algorithm [41], first on the reduced data for the high level and the logarithm of the data for the lower level. The 3D stack of images were reduced to 2D for the purposes of this investigation by aggregating across each image slice in the 3D stack, which simplified the subsequent analysis.

Once the neutrophils had been segmented they were individually labelled. Finally, we obtained the centroid of each segmented neutrophil and also the distance from any neighbours.

#### Tracking

A keyhole model was used to link tracks at contiguous frames, described fully in [31]. To outline the method, the keyhole model predicts the most probable landing position of a neutrophil at sample time 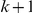 by extrapolating from the positions at samples times 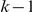 and 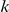. The predicted position of the neutrophil at sample time 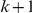 was described by two regions: (i) a narrow wedge (60 deg wide) oriented towards the predicted landing position, and (ii) a truncated circle (300 deg) that complemented the wedge - together they resemble a keyhole. Initially, all segmented neutrophils were examined for possible parent-child relationships using the keyhole model and then a reduced number were formed into a series of tracks. Finally, a post-processing stage was implemented to remove links in tracks that might have been the result of noise and to join sections that had been split in error at the first tracking stage. To perform this correction the keyhole model was used in the backwards time direction.

The tracking algorithm produced estimates of centroid position tracks, defined as coordinates in space,
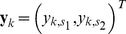
(1)where 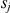, 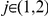 refers to the spatial dimension of the two-dimensional image and where 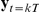 is denoted as 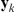, where 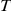 is the sampling time.

The tracking algorithm was applied to both the *in vitro* data set (video 2 from the supplementary material of [30]) as well as the zebrafish data.

### Neutrophil velocity estimation

There are a number of approaches to signal extraction for estimating signal derivatives, which include fitting splines to the signal, frequency domain extraction, e.g. denoising using wavelets, as well as smoothing [42]. The method used here is based on a Taylor-series expansion of the signal in conjunction with Kalman smoothing, which was developed by Fioretti and Jetto [32].

To outline the method, the evolution of the derivatives through time (neutrophil position, velocity, acceleration,…), in each spatial direction 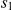 and 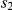, are described by the discrete-time state-space model,
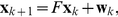
(2)

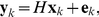
(3)where the state vector at sample time 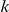, 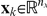, contains up to 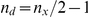 signal derivatives in each spatial direction,
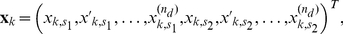
(4)and where 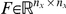 is the state transition matrix, 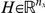 is the measurement matrix, 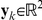 is the vector of neutrophil centroid positions defined in eqn (1) and where 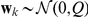 and 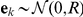 are independent zero-mean Gaussian white noise signals and the further terms of the state-space model are described by [32],
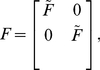
(5)

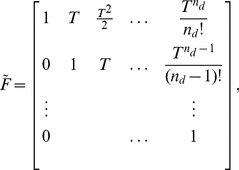
(6)

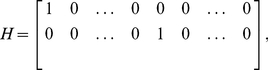
(7)

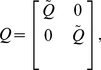
(8)and the elements of the state noise covariance matrix block 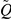 are given by [32],
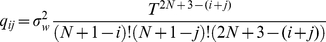
(9)where 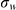 is a tuning parameter describing the power in the state noise.

The state-space model is used in a Kalman smoothing algorithm [33] to obtain the estimate of the signal derivatives. This typically involves using the standard Kalman filter recursions to obtain the filtered state estimate 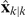, where
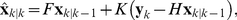
(10)for 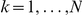, where 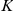 is the Kalman gain. The filter is initialised by defining the normally distributed initial state vector 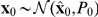. A set of backward recursions are used to obtain the smoothed state estimate 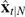, e.g. the Rauch-Tung-Streibel recursions [33],
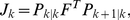
(11)

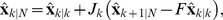
(12)

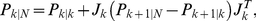
(13)for 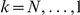, where 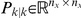 is the filtered state covariance, 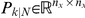 is the smoothed state covariance and 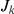 is the smoother gain.

Finally, the neutrophil centroid positions, 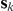, and velocities, 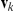, are obtained from collecting the appropriate elements of the smoothed state vector,
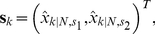
(14)

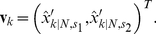
(15)In the implementation of the velocity estimation algorithm the following parameter values were used: state dimension, 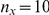; state noise scaling parameter, 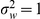; measurement noise covariance, 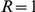; initial state uncertainty, 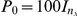, where 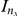 is the identity matrix of dimension 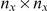; initial state vector, 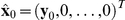, where 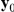 was the initial observation of neutrophil position. Tracks were excluded from the velocity estimation if they had a low number of position samples, 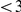 in this case, which reduced the mean number of tracks across fish from 60.3 (std. dev. 20.2) to 50.2 (std. dev. 16.9). Velocity outliers were detected and excluded by first obtaining a histogram of all velocity magnitude estimates, then based on inspection setting an outlier threshold 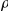, beyond which estimates were classed as outliers, which in this case was set to 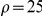 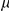
m/min.

### Chemoattractant field estimation

#### Model description

The underlying hypothesis used in this study was that neutrophil velocity is proportional to the gradient of the chemoattractant field,
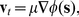
(16)where 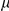 is a proportionality constant, 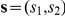 denotes the spatial position, 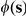 denotes the spatially varying chemoattractant field and 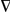 denotes the vector differential operator, hence 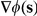 represents the field gradient over space. The task is to estimate the field, 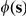, from the velocity of the neutrophil, exploiting the assumed underlying relationship between velocity and field gradient.

If we consider the path of the neutrophil through the vector field of the chemoattractant gradient as a line integral problem, we can relate the spatially varying field gradient to the velocity of the neutrophil as
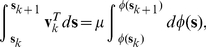
(17)which with 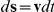 then eqn (17) reduces to
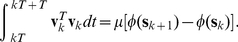
(18)If we assume that 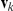 is approximately constant between times 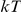 and 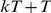 then the LHS of eqn (18) can be written as 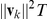, which leads directly to the expression,
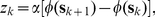
(19)where the constant 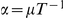 and 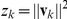. In order to obtain a model-based description of the chemoattractant field, we use a basis function decomposition,
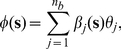
(20)where 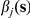 is a basis function and 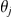 is the associated basis function parameter and 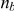 is the number of basis functions. The basis function decomposition of the field leads to the parametric description of the velocity-field gradient relationship by substituting eqn (20) in eqn (19),
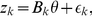
(21)where the constant 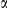 is absorbed into the basis function parameters, the velocity estimate is assumed to be corrupted by independent and identically distributed zero-mean additive Gaussian noise, 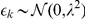, and
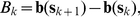
(22)

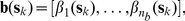
(23)

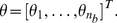
(24)The model is now expressed through eqn (21) in a form suitable for linear estimation of the basis function parameters, 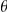. We note that 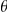 will be non-unique since the proportionality constant 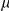 is unknown and influences all elements of 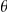. This implies that the estimation procedure for 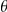 requires the use of regularisation methods or prior probability distributions for the parameters.

The key point to note is that the basis function parameters, 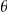, are common to the difference model in eqn (21) *and* the description of the field in eqn (20) - hence, by estimating the parameters using eqn (21) we also obtain the model of the chemoattractant field in the same step. It is this dual use of the basis function parameters that makes this estimation framework particularly elegant and effective.

#### Parameter and field inference

The next stage of the chemoattractant field inference procedure is the estimation of the basis function parameters, 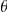, from the model defined in eqn (21). We use a Bayesian method here, where we first place a zero-mean Gaussian prior over the parameters,
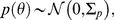
(25)where the variance of the prior is 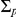. In order to obtain the posterior estimate of the parameters, we require the data-driven maximum likelihood estimate; first, we define the likelihood function,
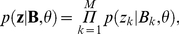
(26)where 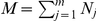 is the total number of neutrophil data points available for driving the model estimation procedure, 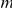 is the number of neutrophil tracks, 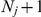 is the number of data points in the 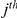 neutrophil track and where all neutrophil track data points are collected in the terms 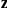 and 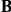, so that from eqn (21),
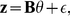
(27)where, for the 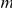 neutrophil tracks,
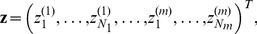
(28)

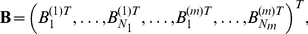
(29)

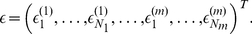
(30)


Using the definition of the likelihood function in eqn (26) we can obtain the posterior estimate of the parameter distribution: noting from Bayes rule that 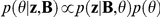 and that the noise term 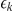 is Gaussian, we obtain the expression for the posterior distribution of the parameters,
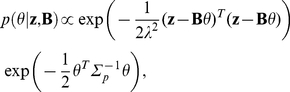
(31)which simplifies to the Gaussian distribution,
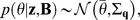
(32)where
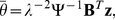
(33)

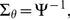
(34)

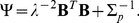
(35)


The identified model of the chemoattractant field can be used to evaluate the field across space, interpolating between observation locations and providing an effective visualisation, where the model prediction of the field, 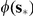 at prediction location 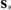, is described by the distribution,
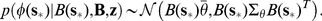
(36)


#### Implementation of the field inference framework

In this investigation we used radial basis functions in the decomposition of the chemoattractant field described in eqn (20), specifically the squared exponential function,
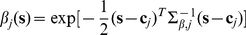
(37)where 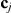 denotes the 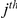 basis function centre and 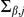 is a diagonal matrix containing basis function widths governing each of the two spatial directions,
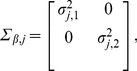
(38)where in this investigation we assumed isotropic basis functions were appropriate, hence 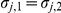.

It is often desirable to represent a model at multiple scales, which captures underlying trends in the data and finer-level detail in separate model components [27], [28]. Hence, a coarse grid of 4 basis functions (2
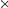
2) were placed and centred at the corners of the image and an additional grid of finer scale basis functions was used (3
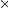
3 for the *in vitro* data and 6
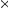
6 for the zebrafish data), where the spacing of centres was reduced to 320 pixels and 100 pixels for the *in vitro* and zebrafish datasets respectively. Basis functions widths were set to half the centre spacing for the zebrafish datasets and equal to the centre spacing for the *in vitro* data. Note that for the purposes of illustrating the zebrafish results the 500
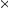
500 pixel grid used in the modelling procedures was re-scaled to the 1000
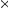
1000 pixel grid of the original image. Boundary conditions were imposed on the model by masking the region outside of the zebrafish. The hyperparameters of the inference model were set to 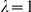 and 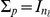, where 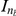 is the identity matrix of dimension 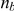.

## References

[1] Porter SL, Wadhams GH, Armitage JP (2011). Signal processing in complex chemotaxis pathways.. Nature Reviews Microbiology.

[2] Swaney KF, Huang C, Devreotes PN (2010). Eukaryotic chemotaxis: a network of signaling pathways controls motility, directional sensing, and polarity.. Annual Review of Biophysics.

[3] Parent CA (2004). Making all the right moves: chemotaxis in neutrophils and dictyostelium.. Current Opinion in Cell Biology.

[4] Nathan C (2006). Neutrophils and immunity: challenges and opportunities.. Nature Reviews Immunology.

[5] Silva MT (2011). Macrophage phagocytosis of neutrophils at inammatory/infectious foci: a cooperative mechanism in the control of infection and infectious inammation.. Journal of Leukocyte Biology.

[6] Singer AJ, Clark RA (1999). Cutaneous wound healing.. The New England Journal of Medicine.

[7] Wu D, Lin F (2011). Modeling cell gradient sensing and migration in competing chemoattractant fields.. PLoS ONE.

[8] Bosgraaf L, Van Haastert PJM (2009). The ordered extension of pseudopodia by amoeboid cells in the absence of external cues.. PLoS ONE.

[9] Neilson MP, Veltman DM, van Haastert PJM, Webb SD, Mackenzie JA (2011). Chemotaxis: a feedback-based computational model robustly predicts multiple aspects of real cell behaviour.. PLoS Biology.

[10] Jilkine A, Edelstein-Keshet L (2011). A comparison of mathematical models for polarization of single eukaryotic cells in response to guided cues.. PLoS Computational Biology.

[11] Soehnlein O, Lindbom L (2010). Phagocyte partnership during the onset and resolution of inammation.. Nature Reviews Immunology.

[12] Elks PM, Loynes CA, Renshaw SA (2011). Measuring inammatory cell migration in the zebrafish.. Methods in Molecular Biology.

[13] Elks PM, van Eeden FJ, Dixon G, Wang X, Reyes-Aldasoro CC (2011). Activation of hypoxiainducible factor-1a (Hif-1a) delays inammation resolution by reducing neutrophil apoptosis and reverse migration in a zebrafish inammation model.. Blood.

[14] Mathias JR, Walters KB, Huttenlocher A (2009). Neutrophil motility in vivo using zebrafish.. Methods in Molecular Biology.

[15] Renshaw S, Loynes C, Trushell D, Elworthy S, Ingham P (2006). A transgenic zebrafish model of neutrophilic inammation.. Blood.

[16] Mathias JR, Perrin BJ, Liu T, Kanki J, Look AT (2006). Resolution of inammation by retrograde chemotaxis of neutrophils in transgenic zebrafish.. Journal of Leukocyte Biology.

[17] Martin J, Renshaw S (2009). Using in vivo zebrafish models to understand the biochemical basis of neutrophilic respiratory disease.. Biochemical Society Transactions.

[18] Renshaw S, Loynes C, Ingham P, Whyte M (2007). Modeling inammation in the zebra_sh: how a fish can help us understand lung disease.. Experimental Lung Research.

[19] Irimia D, Liu SY, Tharp WG, Samadani A, Toner M (2006). Microuidic system for measuring neutrophil migratory responses to fast switches of chemical gradients.. Lab on a Chip.

[20] Jeon NL, Baskaran H, Dertinger SKW, Whitesides GM, Van De Water L (2002). Neutrophil chemotaxis in linear and complex gradients of interleukin-8 formed in a microfabricated device.. Nat Biotech.

[21] Zigmond SH (1977). Ability of polymorphonuclear leukocytes to orient in gradients of chemotactic factors.. The Journal of Cell Biology.

[22] Niethammer P, Grabher C, Look AT, Mitchison TJ (2009). A tissue-scale gradient of hydrogen peroxide mediates rapid wound detection in zebrafish.. Nature.

[23] Olariu V, Coca D, Billings S, Tonge P, Gokhale P (2009). Modified variational Bayes EM estimation of hidden markov tree model of cell lineages.. Bioinformatics.

[24] Dewar M, Kadirkamanathan V, Opper M, Sanguinetti G (2010). Parameter estimation and inference for stochastic reaction-diffusion systems: application to morphogenesis in d. melanogaster.. BMC Systems Biology.

[25] Keller E, Segel L (1971). Model for chemotaxis.. Journal of Theoretical Biology.

[26] Hillen T, Painter KJ (2009). A user's guide to PDE models for chemotaxis.. Journal of Mathematical Biology.

[27] Billings S, Wei H, Balikhin M (2007). Generalized multiscale radial basis function networks.. Neural Networks.

[28] Liu GP, Kadirkamanathan V, Billings SA (1999). Variable neural networks for adaptive control of nonlinear systems.. IEEE Transactions on Systems, Man, and Cybernetics, Part C: Applications and Reviews.

[29] Peterka V (1981). Bayesian system identification.. Automatica.

[30] Tharp WG, Yadav R, Irimia D, Upadhyaya A, Samadani A (2006). Neutrophil chemorepulsion in defined interleukin-8 gradients in vitro and in vivo.. Journal of Leukocyte Biology.

[31] Reyes-Aldasoro C, Akerman S, Tozer GM (2008). Measuring the velocity of uorescently labelled red blood cells with a keyhole tracking algorithm.. Journal of Microscopy.

[32] Fioretti S, Jetto L (1989). Accurate derivative estimation from noisy data: a state-space approach.. International Journal of Systems Science.

[33] Kailath T, Sayed AH, Hassibi B (2000). Linear Estimation..

[34] Dewar M, Scerri K, Kadirkamanathan V (2009). Data-driven spatio-temporal modeling using the integro-difference equation.. IEEE Transactions on Signal Processing.

[35] Scerri K, Dewar M, Kadirkamanathan V (2009). Estimation and model selection for an IDE-based spatio-temporal model.. IEEE Transactions on Signal Processing.

[36] Coca D, Billings S (2002). Identification of finite dimensional models of infinite dimensional dynamical systems.. Automatica.

[37] Freestone D, Aram P, Dewar M, Scerri K, Grayden D (2011). A data-driven framework for neural field modeling.. NeuroImage.

[38] Wikle CK, Holan SH (2011). Polynomial nonlinear spatio-temporal integro-difference equation models.. Journal of Time Series Analysis.

[39] McDonald B, Pittman K, Menezes GB, Hirota SA, Slaba I (2010). Intravascular danger signals guide neutrophils to sites of sterile inammation.. Science.

[40] Burt P, Adelson E (1983). The Laplacian pyramid as a compact image code.. IEEE Transactions on Communications.

[41] Otsu N (1975). A threshold selection method from gray-level histograms.. Automatica.

[42] Young P, Pedregal D (1999). Recursive and en-bloc approaches to signal extraction.. Journal of Applied Statistics.

